# Analysis on Seismic Performance of Steel-Reinforced Concrete-Filled Circular Steel Tubular (SRCFST) Members Subjected to Post-Fire

**DOI:** 10.3390/ma15062294

**Published:** 2022-03-20

**Authors:** Yi Han, Yanhong Bao

**Affiliations:** 1 College of Water Conservancy and Hydropower Engineering, Gansu Agricultural University, Lanzhou 730070, China; 2 Key Laboratory of Disaster Prevention and Mitigation in Civil Engineering of Gansu Province, Lanzhou University of Technology, Lanzhou 730050, China; 3 School of Civil Engineering, Qinghai University, Xining 810016, China; cebaoyh@163.com

**Keywords:** steel-reinforced concrete-filled circular steel tubular (SRCFST) members, post-fire, seismic performance, parameter analysis

## Abstract

Steel-reinforced concrete-filled steel tubular (SRCFST) columns have a great development prospect in engineering practice due to their high load-bearing capacity, good ductility, and energy consumption capacity. This paper established the post-fire seismic analysis model of SRCFST with a circular-cased H section using the sequential coupled thermal-stress method by ABAQUS. The *P*-*Δ* curve, stiffness, ductility, and energy dissipation were calculated. Then, the post-fire seismic performance of CFST members was compared while keeping the total steel ratio constant, and it revealed that the SRCFST had superior ductility to CFST. Finally, the ductility coefficient and skeleton curve were parametrically evaluated. The results of the study showed that the effects of heating time (*t_h_*), axial compression ratio (*n*), slenderness ratio (*λ*), and steel tube ratio (*α_t_*) on the skeleton line of SRCFST columns are more significant; the axial compression ratio (*n*), slenderness ratio (*λ*), and steel tube ratio (*α_t_*) have a negative influence on the ductility subjected to post-fire.

## 1. Introduction

**Figure 1:**
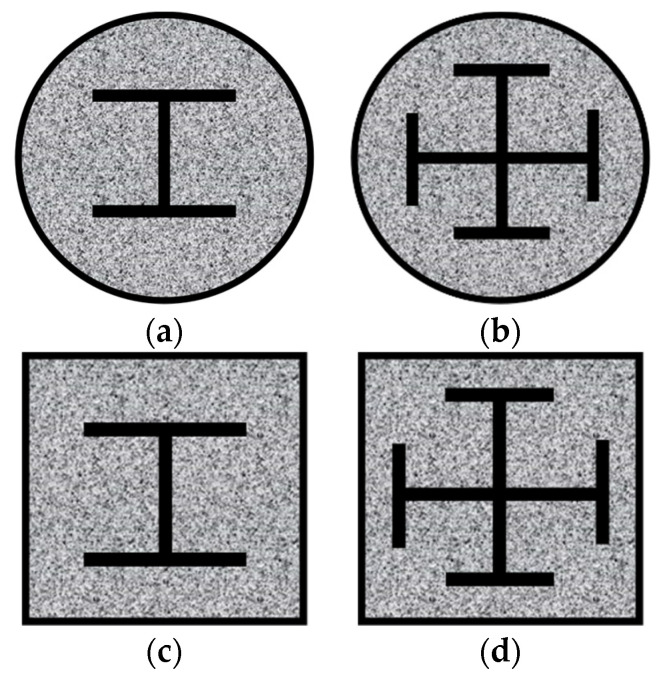
Section type of SRCFST: (**a**) Circular-cased I section; (**b**) Circular-cased crossed section; (**c**) Square-cased I section; (**d**) Square-cased crossed section.

Due to its outstanding mechanical qualities, concrete-filled steel tube (CFST) structures have been used in an extensive range of high-rise and super-tall constructions. However, with the development of the building structure toward the direction of heavy load, high rise, long span, and adverse conditions, it is not easy to meet the use requirements of some mega-high-rise buildings only with the section structure of plain concrete filled in the steel tube. As a result, many more designs are added to the core concrete-filled steel tube’s external and internal surfaces, such as adding composite-reinforced concrete columns outside the steel tube and designing new CFST columns with variable section forms of steel bars inside the core concrete. The concrete-filled steel tubular column with built-in steel, also known as a steel-reinforced concrete-filled steel tubular (SRCFST) column, is a novel composite column constructed by inserting steel into an empty steel tube and pouring concrete into the steel tube [1]; the primary sectional forms of the components are shown in Figure 1. This new type of composite column is first proposed in the EN 1994-1-1 [2]. It combines the benefits of both SRC and CFST columns, and it outperforms both in several areas, including bearing capacity, energy dissipation capacity, and ductility. As a result, this new composite column has great potential in engineering [3,4].

In recent years, scholars have conducted abundant experimental studies and theoretical analyses on the mechanical properties of SRCFST columns at ambient temperature. Wang et al. [3,4,5,6] conducted an experimental study on axially compressed SRCFST members and discovered that adding profiled steel to CFST effectively improved the load capacity and ductility of column members, as well as a parametric analysis of the factors affecting the load capacity and ductility of such members, in order to propose a load capacity formula for SRCFST axially compressed short columns. Xu et al. [7] carried out finite element simulations of SRCFST axial compression short columns to analyze their damage morphology. Ding et al. [8] established a bearing capacity equation for short axially compressed SRCFST columns with the circular section based on the limit equilibrium theory. Based on the theoretical method of tangential modulus, Zhu et al. [9,10] investigated the SRCFST axial compression long column experimentally and derived a simplified formula for the elastic–plastic bearing capacity and the critical slenderness ratio, and they proposed a practical calculation formula for the bearing capacity of this member. Wang et al. [11] did a one-way bias test on the SRCFST column and analyzed the force mechanism and damage pattern. He et al. [12] researched eccentricity tests on self-compacting high-strength concrete with internal steel sections and found that the eccentricity was the most significant factor affecting the bearing capacity of such members. Cai et al. [13] quantitatively analyzed the force performance of SRCFST under eccentric loading and discovered that the Eurocode 4 model greatly overestimated the load-carrying capacity of this type of member, and they presented a prediction model for this type of member’s load-carrying capacity. Wang et al. [14] completed a finite element analysis of the bending performance of SRCFST and observed that the internally mated sections reduced the neutral axis movement and the expansion of concrete bending cracks. Zhao and Wang [15] developed an analytical process for steel tube-integrated steel high-strength concrete compression bending elements based on their expertise of the strip method. The mechanical properties of SRCFST members subjected to shear [16] and torsion [17] have also been investigated successively. In addition to the simple stresses of the SRCFST members, Wang et al. [18,19,20] investigated the mechanical properties under composite stresses of compression–torsion and compression–bending–shear by means of test methods and numerical calculations. Using ABAQUS finite elements, Xu et al. [21] calculated the hysteresis performance of SRCFST members under reciprocating loads and discovered that due to the profile steel, the stiffness, peak load, and deformation performance of such members were superior to that of standard CFST columns. The dynamic response of SRCFST members under transverse impact loading was studied experimentally in the literature [22,23], with the section of steel, impact velocity, and impact direction all being taken into account, and the results showed that such columns can absorb a large amount of input energy and have better impact resistance.

Fire, as a major calamity, has caused serious life hazards and property damage to human society, so the fire resistance of SRCFST components has been explored. Han et al. [24,25,26] conducted a preliminary investigation of the fire behavior of SRCFST columns exposed to uniform, non-uniform, and full-range fires. Meng et al. [27] numerically carried out the calculation of the residual bearing capacity of SRCFST after ISO834 standard fire and proposed a prediction formula for the residual strength index of SRCFST columns with the square section under different fire exposure modes. This type of component has been tested under uniform and non-uniform fire by Mao et al. [28] and Fqma et al. [29]. After the fire, except for the collapsed buildings, the other buildings that have not collapsed need to have their mechanical properties evaluated. It is especially essential to evaluate the post-fire seismic behavior of buildings or components to determine whether the bearing capacity and stiffness of the building structure fulfill the original design criteria after the fire [30]. At present, there are fruitful exploration results on the building structures’ fire resistance, and the study on the fire resistance of components has been developed to the overall system [31,32]. In contrast, there are fewer study results on the behavior of building structures after exposure to fire. Initially, the exploration of the post-fire behavior of structures mainly focused on the constitutive relationship of materials and the mechanical properties of components. Han et al. [33] and Huo et al. [34] investigated the bearing capacity and energy dissipation capacity of CFST column–steel beam cruciform joints under horizontal reciprocating load after fire. Song [35] did tests of the temperature field and the post-fire mechanical properties of CFST column–steel beam joints and SRC column–SRC beam joints considering the full-range fire, and they put forward the practical calculation method of the moment–rotation relationship and the residual stiffness of joints. Liu et al. [36] explored the seismic performance of an RC shear wall subjected to post-fire. Yaqub et al. [37,38] carried out the seismic behavior of RC columns strengthened with CFRP after fire. In these papers [30,39,40,41], the seismic performance of SRC columns after fire is studied experimentally, and the post-fire finite element study model of the component is proposed; through numerical analysis, a simplified calculation method of the bearing capacity of SRC after fire is proposed. After fire damage, Li et al. [42] carried out an experimental investigation on the seismic behavior of SRC beam–column joints, and the findings show that as the axial compression ratio increases, the stiffness and strength of the joints improve. However, the ductility coefficient decreases, and the hysteretic performance of SRC beam–column joints remains good. Wang et al. [43] investigated the seismic performance of SRC frame structures after exposure to high temperatures, taking into account the effect of fire temperature rise and fall, and they developed an FE analysis model of the temperature field and post-fire seismic performance of SRC frame structures.

In summary, there is little research on the post-fire seismic performance of SRCFST members so far, which hinders the promotion of the use of such high-performance members, so this paper provides a preliminary investigation of the seismic performance of SRCFST columns subjected to ISO-834 standard fire, following fire and at room temperature, comparing and assessing the load–deformation hysteresis connection, skeleton curve, stiffness degradation, ductility, and other seismic performance criteria of the members. Finally, the main affecting parameters of skeleton line and ductility coefficient after fire were parametrically analyzed. The findings could serve as a guide and foundation for safety assessments and fire-reinforcement repairs as well as help to improve the design theory of SRCFST members.

## 2. Finite Element Modeling

As a result of the combined effects of high temperature and reciprocating load, the seismic performance model after fire is the coupling calculation of temperature field and mechanical field. The sequential coupled thermal-stress method has been widely used because of its good convergence, simulation, and efficiency. The seismic performance calculation model of a steel-reinforced concrete-filled steel tubular (SRCFST) column after fire is established by ABAQUS (ABAQUS 6.14-4, Dassault SIMULIA, Johnston, RI, USA).

### 2.1. Temperature Field Calculation Model

#### 2.1.1. Thermal Parameters

The most crucial item to assure the correctness of the temperature field model is to accurately identify the thermal properties of steel and concrete, which primarily include thermal conductivity, specific heat, and thermal expansion coefficients. Scholars have studied the thermal performance of steel and concrete extensively. Han [44] selected the thermal parameters of steel and concrete given by Lie [45] when simulating the temperature field of CFST components. The obtained temperature–time curve is in acceptable simulation with the experiment, so the same thermal model is used in this paper.

#### 2.1.2. Contact Thermal Resistance

Complete heat transfer is assumed while calculating the temperature field, and the contact thermal resistance between concrete and steel is neglected. Between a steel tube and concrete, as well as between concrete and profiled steel, a tie-binding constraint is used. Different materials have the same temperature at the unit nodes at the same geometric position. The ISO-834 standard heating curve was used to calculate the uniform fire surface. The radiation coefficient was 0.5, and the convection coefficient was 25 W/(m^2^ °C). The 8-node brick elements were used in the steel tube, concrete, and profiled steel (C3D8R). The global seeds were set to mesh the components through the structural meshing technology, and the seeds on edge were set to mesh the local area.

### 2.2. Calculation Model of Post-Fire Seismic Performance

#### 2.2.1. Material Properties

The temperature after fire is related to the historical maximum temperature experienced by the material. The maximum temperature under fire is extracted as the over fire temperature of the material after fire. The constitutive model of concrete material after fire selects the concrete stress–strain relationship curve proposed by Lin [46], and the stress–strain relationship model of a concrete-filled steel tubular at the post-high-temperature stage is obtained by modifying the peak stress and peak strain of the stress–strain relationship model of CFST at ambient temperature. The steel is designed using the double broken-line model with the following specific expression:

(1) $$\sigma =\left\{ \begin{array}{l} E_{s}(T_{\max})\varepsilon \;\;\;\;\;\;\;\;\;\;\;\;\varepsilon \leq \varepsilon_{y}(T_{\max})\\ f_{y}(T_{\max})+E_{s}^{’}(T_{\max})[\varepsilon -\varepsilon_{y}(T_{\max})]\;\;\;\varepsilon >\varepsilon_{y}(T_{\max}) \end{array}\right.$$

The yield limit after the high temperature is determined according to the following formula:

(2) $$f_{y}(T_{\max})=\left\{ \begin{array}{l} f_{y}\;\;\;\;\;\;\;\;\;\;\;\;\;\;\;\;\;\;\;\;\;\;T_{\max}\leq 400\;{}^{\circ}C\\ f_{y}[1+2.23\times 10^{-4}(T_{\max}-20)-5.88\times 10^{-7}(T_{\max}-20{)}^{2}]\;T_{\max}>400\;{}^{\circ}C \end{array}\right.$$

*T*_max_ is the highest temperature in history,

Elastic stage: $E_{\mathrm{sp}}(T_{\max})=E_{s}=2.06\times 10^{5}$ MPa

Strengthening section: $E_{\mathrm{sp}}{}^{’}(T_{\max})=0.01E_{s}(T_{\max})=2.06\times 10^{3}$ MPa

#### 2.2.2. Geometric Model

The steel tube, profiled steel, concrete, and rigid plate are utilized as solid elements (C3D8R). The mesh division should be consistent with the temperature field in order to identify the temperature of each node accurately. In the SRCFST, the interface between the steel tube and concrete, as well as the interface between the concrete and profiled steel, uses “surface to surface” contact; tangential and normal contact surfaces were considered, with the tangential using the Cullen friction model and the normal using the hard contact model. A “tie” constraint is applied in the rigid plate and other components.

#### 2.2.3. Loading Procedure

**Figure 2:**
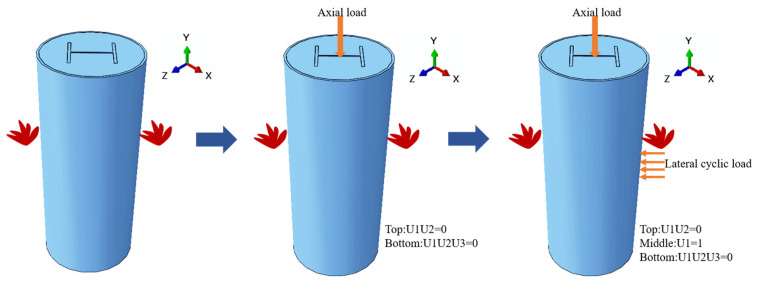
Numerical calculation steps and loading illustration.

**Figure 3:**
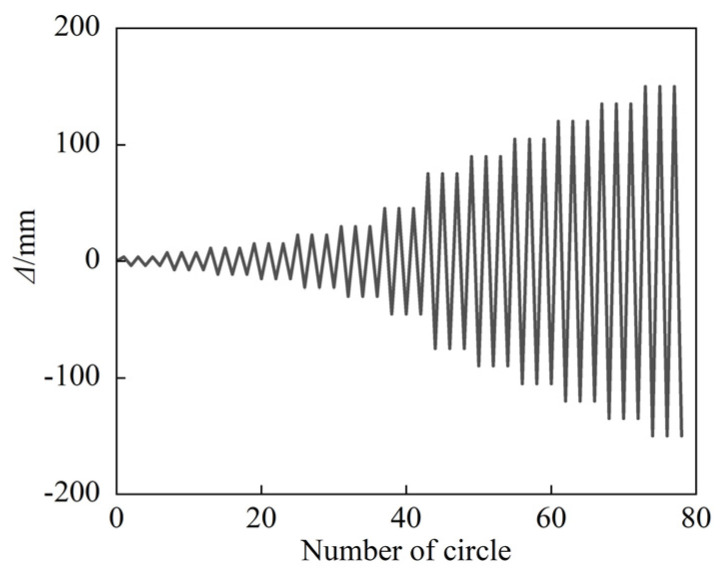
Cyclic loading protocol.

There are three steps to the FE modeling process. First, the column members are heating; then, the column top and bottom are hinged first, with a constant axial load applied at the top of the column. Finally, the reciprocal lateral displacement load is applied at the middle-span of the specimen. Figure 2 depicts a schematic diagram of boundary conditions. The displacement protocol from the JGJ/T 101–2015 configuration [47] was used in the study, and the target displacement levels were 0.25*Δ*_y_, 0.5*Δ*_y_, 0.75*Δ*_y_, 1*Δ*_y_, 1.5*Δ*_y_, 2.0*Δ*_y_, 3.0*Δ*_y_, 5.0*Δ*_y_, 7.0*Δ*_y_, and 8.0*Δ*_y_, in which *Δ*_y_ is the yield displacement of the specimen, and there are three cycles per level, respectively. The loading amplitude is shown in Figure 3.

### 2.3. Verification of Numerical Calculation Model

**Figure 4:**
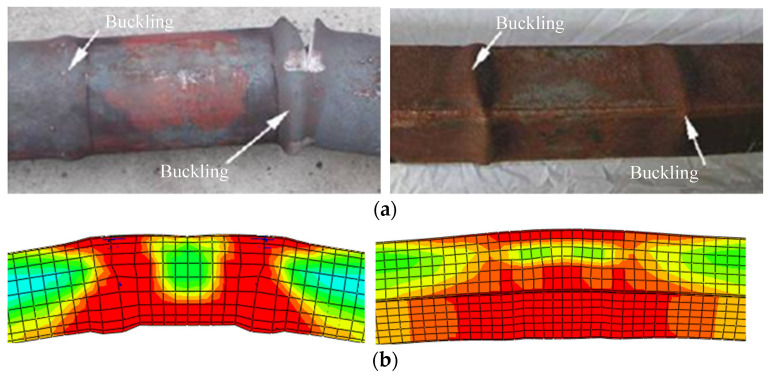
Failure morphology of typical components by experiment and numerical simulation: (**a**) Failure morphology of experiment columns; (**b**) Failure morphology of numerical calculation columns.

**Figure 5:**
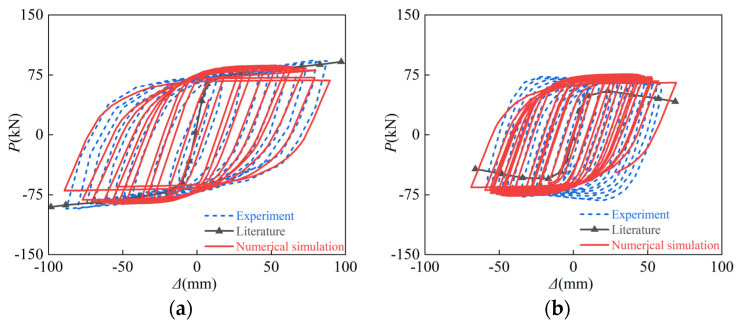

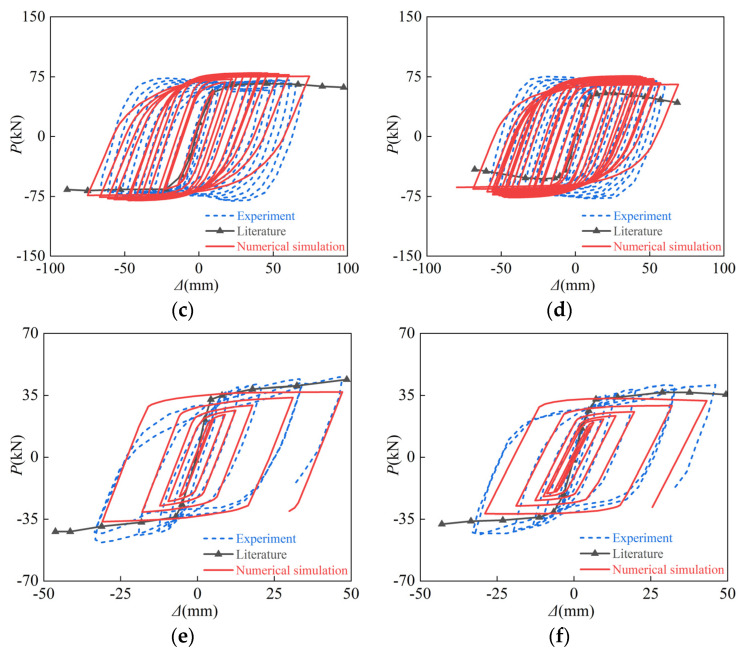
*P-Δ* hysteresis curves after exposure to fire: (**a**) CF1; (**b**) CF2; (**c**) CF3; (**d**) CF5-1; (**e**) SF1; (**f**) SF2-1.

So far, there are no test data on the post-fire seismic performance of SRCFST members; the CFST member tests in the document [46] were selected to verify the accuracy of the finite element model in this research. Table 1 is the test specimen parameters for the hysteretic behavior of CFST after fire. Figure 4 shows the comparison of failure modes of a concrete-filled circular steel tubular column and concrete-filled square steel tubular column. Numerical calculation buckling is compatible with the test, and the failure mode is likewise identical. Figure 5 compares the experimental and numerical results of the *P-Δ* curve of CFST members after fire as well as the skeleton line of FE calculation in the literature [46]. It can be seen that the peak load of the hysteresis curve and the slope of the unloading phase curve are not significantly different from the test, and the peak load calculated in the literature [46] is slightly low. In general, the numerical calculations in this paper accord well with the test, indicating that the model is reliable and applicable.

## 3. Numerical Calculation Results and Discussion

To investigate the post-fire hysteretic behavior of concrete-filled circular steel tubular compression and bending members with H-section steel after fire, we refer to the actual project typical size. According to GB 50936-2014 [48], JGJ138-2001 [49], and other requirements, a standard member is designed; the details of its parameters are shown in Table 2. The meaning of each parameter is the same as that in the literature [50]; the profiled steel is used for HW175 × 175 × 7.5 × 11.

### 3.1. Temperature Field Analysis

**Figure 6:**
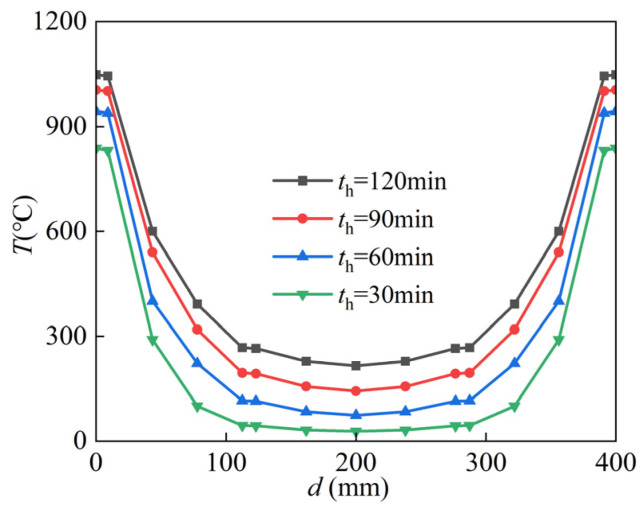
Temperature–depth curves.

Figure 6 illustrates the temperature variation at different positions in the section of the SRCFST member at different moments, where *d* is the depth along the diameter.

**Figure 7:**
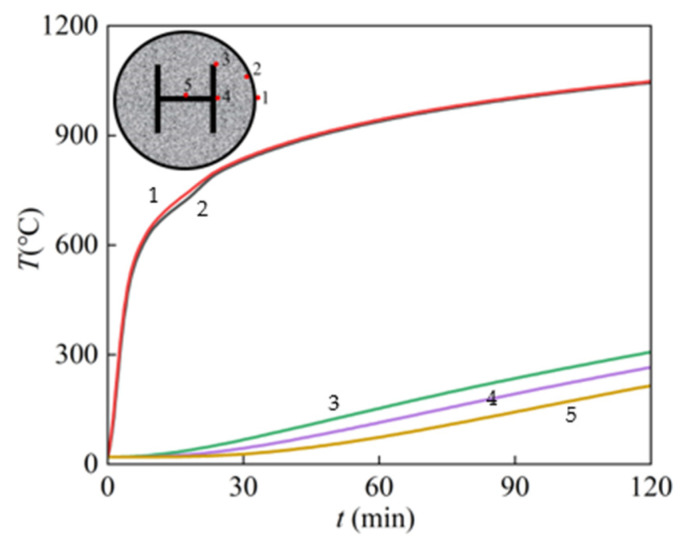
Temperature–time curves.

Figure 7 presents the temperature–time relationship curves for each characteristic point of the SRFCFST column section, where point 1 is the outer surface of the steel tube, point 2 is the outer surface of the concrete, point 3 is the point on the edge of the section flange, point 4 is the midpoint of the upper surface of the section flange, and point 5 is the midpoint of the outer surface of the web of the section.

The temperature of the SRCFST member section is symmetrically distributed along the diameter; as seen in the above two figures, the closer to the center of the circle, the lower the temperature, and the more noticeable the temperature lag phenomena.

The surface temperature of the steel pipe rises rapidly at the beginning of the fire; when the heating time is 120 min, the temperature at point 1 is 1048 °C, and the temperature at point 2 is 1044 °C. Here, it can be seen that the temperature difference between the outer surface of the concrete and the outer surface of the steel tube is very small. The temperature of point 3 on the edge of the profile flange is 307 °C, while that of point 4 in the middle of the flange is 265 °C, and that of point 5 on the web is 215 °C. This is because the concrete is thermally inert and acts as a natural fire protection layer for the profile steel, reducing the temperature on the profile surface and enabling the strength and ductility to shine.

**Figure 8:**
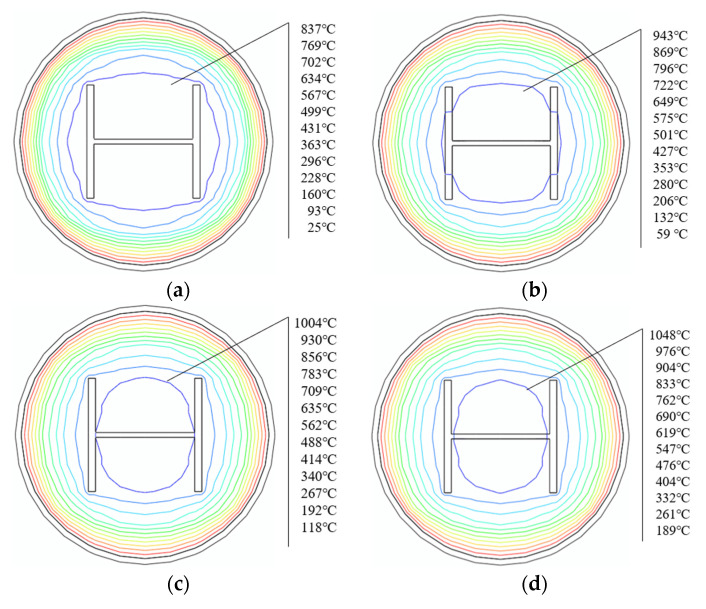
Temperature distribution of section at different times: (**a**) 30 min; (**b**) 60 min; (**c**) 90 min; (**d**) 120 min.

The temperature distribution of the SRCFST member of the cross-section at different points in the midspan is shown in Figure 8, and the concrete exhibits temperature thermal inertia, with a large temperature gradient at the edge of the section and a closer maximum temperature in the middle. The temperature of the cross-section can develop faster when the heating time is less than 30 min, and when the heating time is more than 90 min, the temperature increase in the cross-section becomes slow. This is because with the prolonging of the heating period, the temperature is transferred to the inside of the section through the thermal conductivity of the peripheral steel tube, and the temperature of the member increases. However, the thermal conductivity of the steel decreases with increasing temperature; when the temperature exceeds a certain limit, the thermal conductivity of steel becomes almost constant [44]. The regulation can also be derived in Figure 7.

### 3.2. Failure Mode

**Figure 9:**
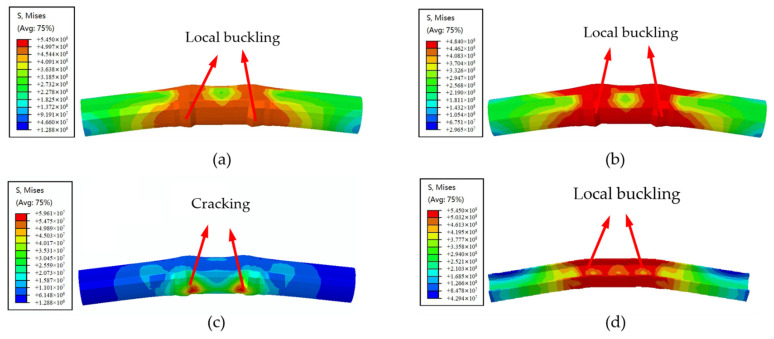
Failure mode of specimen SRCFST: (**a**) SRCFST; (**b**) Tube; (**c**) Concrete; (**d**) Profiled steel.

The numerical simulation model for SRCFST seismic performance after fire has been established using the above-mentioned modeling method. Figure 9 indicates the failure mode of typical members and components. It can be seen that the failure mode of reciprocating loading after fire is similar to CFST. Compression buckling occurs in the column, and during subsequent unloading and reverse loading, the buckling part is flattened again and causes the compression buckling on the other side. The buckling phenomena became more severe as reciprocal displacement increased, yet this type of member still has a high bearing capacity.

### 3.3. Hysteresis Curves and Skeleton Curves

**Figure 10:**
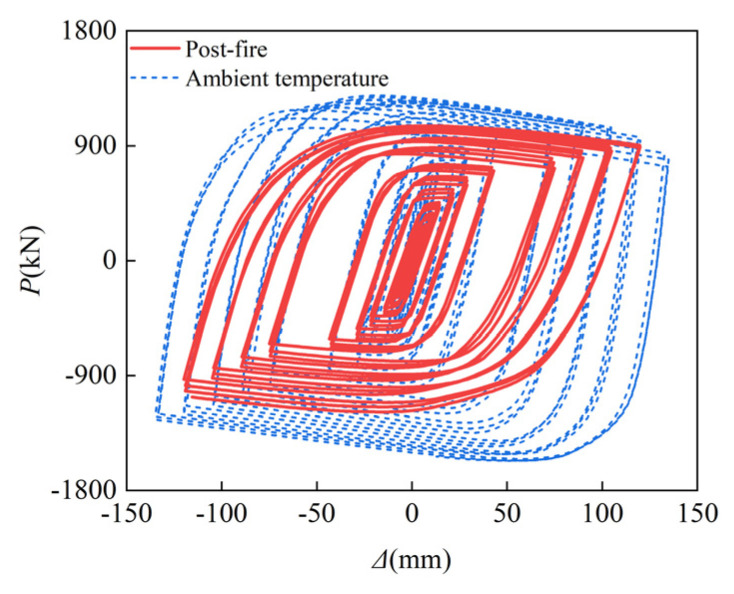
Hysteresis curves of *P-Δ* relations.

Seismic performance refers to the bearing capacity, ductility, energy dissipation capacity, and other properties of building structures and components. These indicators are critical in determining how well buildings perform in large earthquakes. Therefore, the hysteresis curve after fire is calculated, and that at room temperature is also presented. As shown in Figure 10, the shape of the hysteresis loop of the SRCFST column is essentially the same as at room temperature, and while it is not as complete as the corresponding column at ambient temperature, there is no obvious pinching and shrinkage, and it still performs well in seismic evaluations.

**Figure 11:**
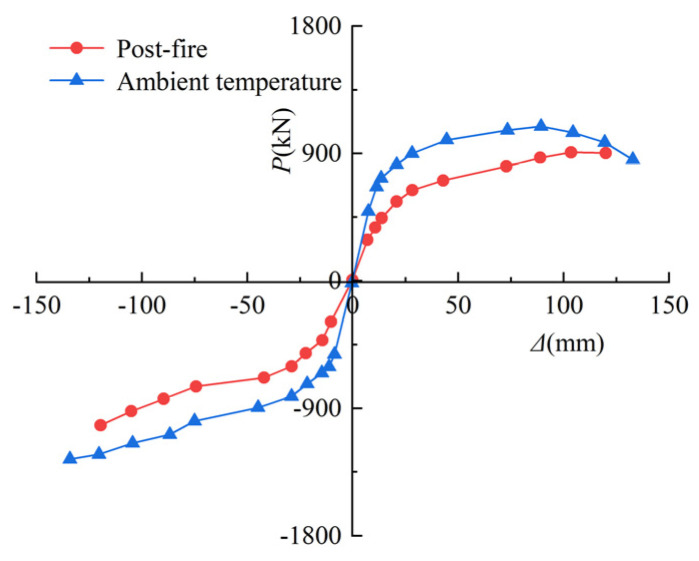
Skeleton curves of *P-Δ* relations.

The skeleton curve is obtained by connecting the peak points of the first cycle on each loading level to generate the *P*-*Δ* relations envelope curve, which may describe the variable characteristics of strength and stiffness in the process of reciprocating loading. Figure 11 depicts the skeleton curve of the SRCFST in various situations; it should be noted that the different rules of the skeleton curves of the column are essentially similar in both cases, except that when the effect of fire is considered, the ultimate bearing capacity of the members is reduced, and the corresponding elastic stiffness is also reduced.

### 3.4. Ductility

**Figure 12:**
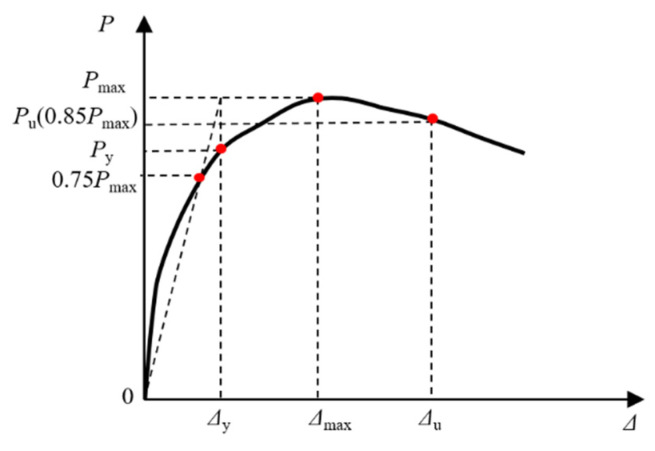
Characteristic points of *P-Δ* relations.

The deformation capacity of a member when subjected to a load, as well as the displacement, is referred to as ductility [47]. The ductility coefficient (*μ*) is defined as follows [47]:

(3) $$\mu =\frac{\Delta_{u}}{\Delta_{y}}$$

where *Δ*_u_ is the failure displacement and *Δ*_y_ is the yield displacement; the accuracy of the yield displacement directly affects the reliability of the ductility coefficient. The yield displacement was calculated adopting the method proposed by Park et al. [51]. Figure 12 shows the envelope curve, where *P*_y_, *P*_max_, *Δ*_max_, and *P*_u_ denote the yield load, maximum strength, maximum displacement, and failure load, respectively. Table 3 shows a considerable difference in the maximum load and ductility of this column unfired and post-fire, with the maximum load (*P*_max_) of post-fire reduced by 18%, *Δ*_u_ decreased by 10%, and ductility decreased by 41.9% when compared to the unfired component. However, the *Δ*_y_ increased by 40% after fire. This is because under natural cooling conditions, the steel yield strength and ultimate strength reduction coefficient tend to decrease significantly as the maximum overfire temperature rises, while elongation after fracture rises [52].

### 3.5. Stiffness Degradation

**Figure 13:**
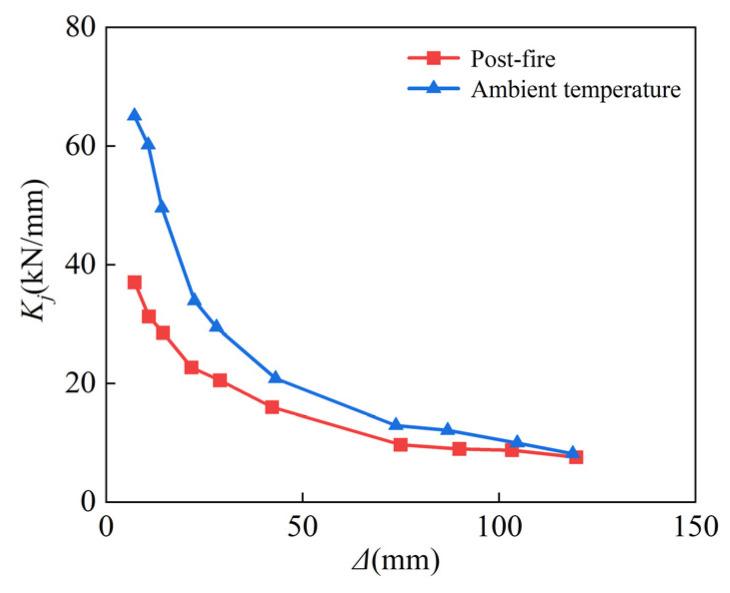
Stiffness degradation.

The degree of damage to the specimen under cyclic loading is reflected in the deterioration of stiffness, which is expressed using the secant stiffness, which is computed using the following equation [47]:

(4) $$K_{j}=\frac{|+P_{j}|+|-P_{j}|}{|+\Delta_{j}|+|-\Delta_{j}|}$$

where *P_j_* is the load value of the positive and negative peak point under the first cycle of level *j*, and *Δ_j_* is the corresponding displacement [53]. The calculation results are shown in Figure 10; owing to the effect of temperature, the stiffness of the column reduced with the degradation of material properties, where the displacement values were 7 mm, 43 mm, and 119 mm, respectively. The stiffness of the column deteriorated with the decline of material qualities, as shown in Figure 13, due to the influence of temperature, where the displacement was 7 mm, 43 mm, and 119 mm, respectively. The post-fire stiffness degradation rate of the specimen was 43.1%, 23.3%, and 7.5%, respectively, when compared to unfired, demonstrating that the stiffness difference between the two working conditions gradually decreases as the lateral displacement increases, because the concrete cracks and crushes as the reciprocal displacement increases.

### 3.6. Energy Dissipation

**Figure 14:**
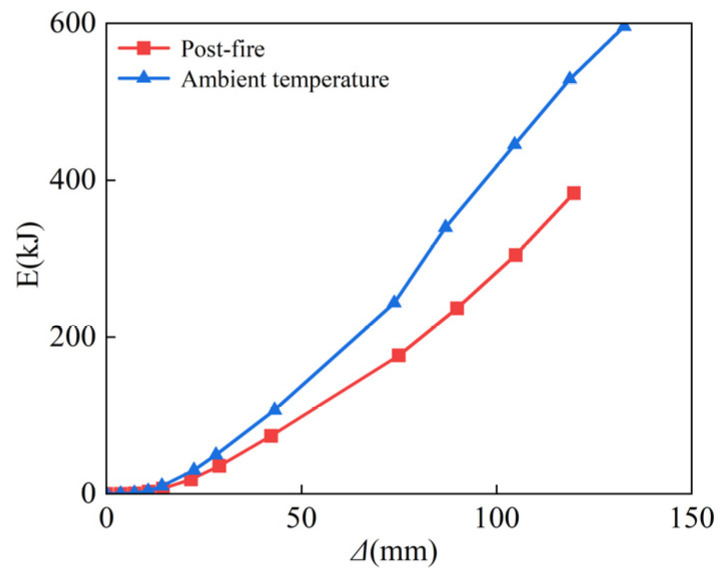
Energy dissipation.

The area of the load–deformation hysteresis curve should be used to calculate the energy dissipation capability of the specimen [47]. Figure 14 shows that the energy dissipation of the SRCFST column is large at room temperature, while post-fire, it is minor, at the beginning of loading. When the displacement is 7 mm, 43 mm, 73 mm, and 119 mm, respectively, the energy consumption decreased by 14.4%, 30.6%, 27.5%, and 27.4%, respectively; thus, it can be proved that the variability enhanced with the increase in displacement before stabilizing. This is because the lateral displacement is minor at the start of loading, and the difference in energy dissipation capacity of the column between the two operating situations at ambient temperature and after fire is not significant. However, the energy dissipation of the post-fire part is smaller than that at ambient temperature as the transverse displacement increases, which is due to the deterioration of material characteristics subjected to post-fire.

### 3.7. Comparison of Post-Fire Seismic Performance of SRCFST and CFST Columns

**Figure 15:**
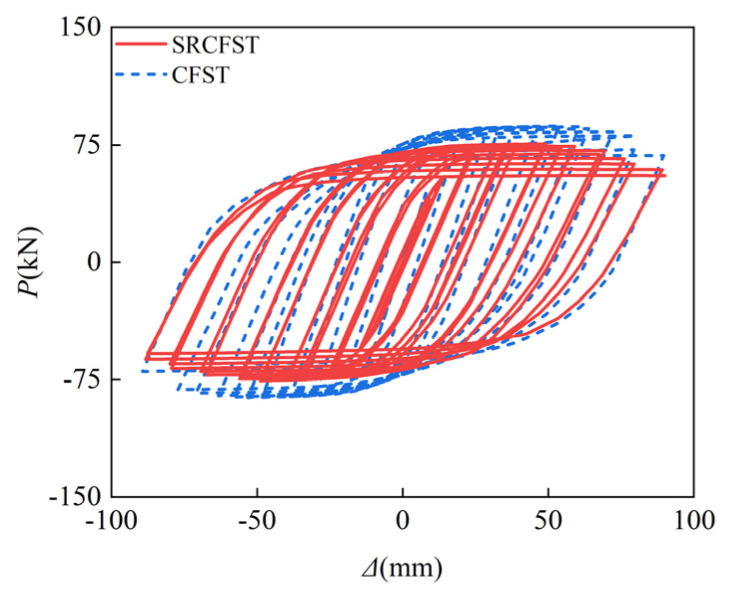
*P*-*Δ* hysteretic curves.

**Figure 16:**
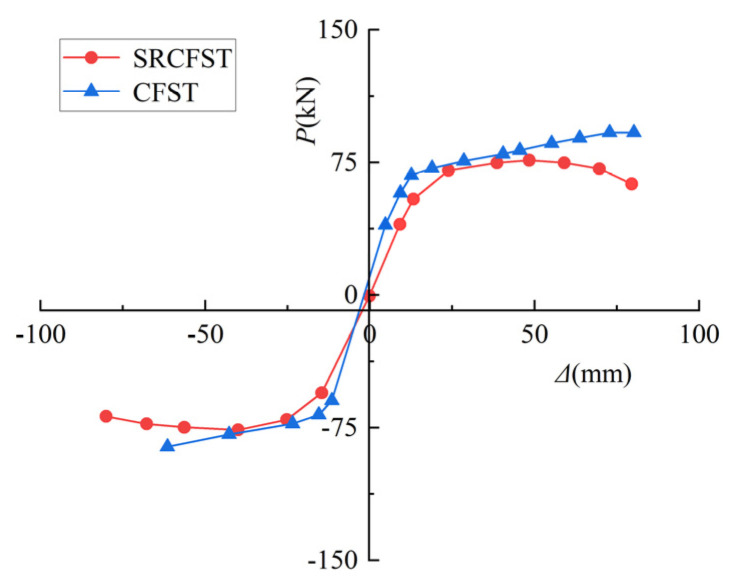
*P*-*Δ* skeleton curves.

This paper used the CF1 specimen from the literature [46] as the basis for comparing the post-fire seismic performance of the steel-reinforced concrete-filled steel tubular column without any structure inside the tube. It ensured that the total cross-section steel ratio remained unchanged, and it placed part of the peripheral steel tube in the form of profiled steel in the core concrete to form an SRCFST column, while all other parameters remained unchanged. The profiled steel is HZ80 (*H* = 80 mm, *B* = 50 mm, *t*_w_ = 3.3 mm, *t*_f_ = 5.2 mm), and the steel profiled ratio is 0.06. Figure 15 and Figure 16 show the comparison results of the hysteresis curve under reciprocating load after exposure to fire and the skeleton curve for both types of columns. Since part of the steel tube is embedded as steel sections in the core concrete, the thickness of the steel tube thins out, reducing the constraint impact on the specimen. The peak stress is also lowered post-fire, but the reduction is minor. Table 4 shows the results of the ductility coefficient calculation for the two types of members. It can be concluded that the *P*_max_ of SRCFST is reduced by 14.3%, the *Δ*_max_ is increased by 12.6%, and the *Δ*_u_ is increased by 11.2% when compared to CFST, indicating that the ductility of SRCFST has increased.

**Figure 17:**
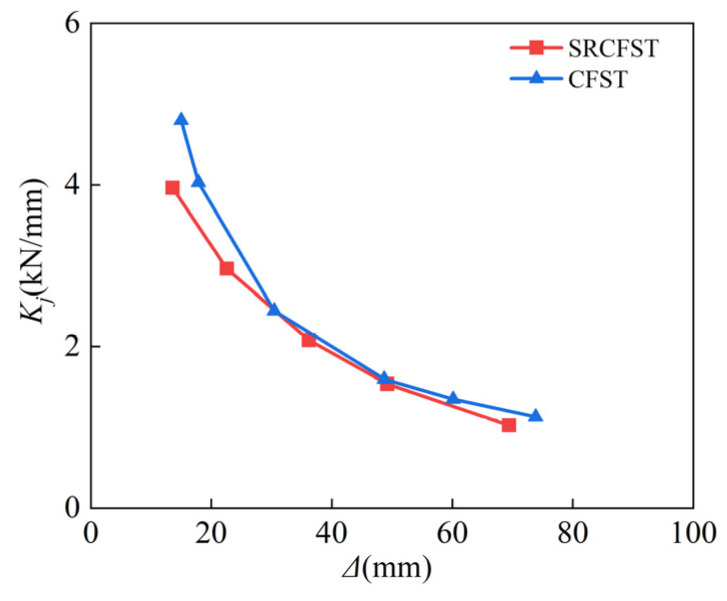
Comparison of stiffness degradation.

Figure 17 shows a comparison of the secant stiffness of the two members after fire; in general, the stiffness of CFST is greater than that of SRCFST. However, at the early stage of loading, when *Δ* < 30 mm, the stiffness degradation rate of the CFST is 49.1%, while that of the SRCFST is 32.2%, implying that the deterioration of the material of the peripheral steel tube affected by high temperature leads to the stiffness of the CFST decreasing, but the SRCFST’s stiffness degradation rate is relatively lower because of the protective effect of concrete on the internal profiled steel. When the reciprocal displacement is 30–50 mm, the secant stiffness degradation rate of the two types of members is equal. However, when the reciprocal displacement is over 50 mm, the *K_j_* of SRCFST is greater than that of CFST. This is because as the reciprocal displacement increases, the internal steel section buckles, while the peripheral steel tube wall thickness is thinner. Although the steel material properties have recovered after exposure to fire, the restraint effect is not as good as CFST. As a result, embedding the steel section within the core concrete improves the ductility of the member after a fire and promotes seismic resistance. To give the advantages of the SRCFST columns, the proportion of the steel ratio of the profiled steel and steel tube is significant. The influence of the profiled steel ratio can be specifically investigated in future research, and a reasonable ratio can be proposed for reference in the actual project.

## 4. Parameter Analysis of Seismic Performance

In order to research the influence of the main parameters of the SRCFST column after exposure to fire, such as heating time (*t_h_*), axial compression ratio (*n*), slender ratio (*λ*), profiled steel ratio (*α_s_*), steel tube ratio (*α_t_*), concrete cubic compressive strength (*f*_cu_), yield strength of steel tube (*f*_yt_), yield strength of profiled steel (*f*_ys_), and protective layer thickness (*a*), a range of parameters commonly used in engineering were selected. As shown in Table 5, the skeleton curves and the ductility coefficients were computed to catch the changing patterns.

### 4.1. Parameters Analysis of Skeleton Curve

#### 4.1.1. Heating Time

**Figure 18:**
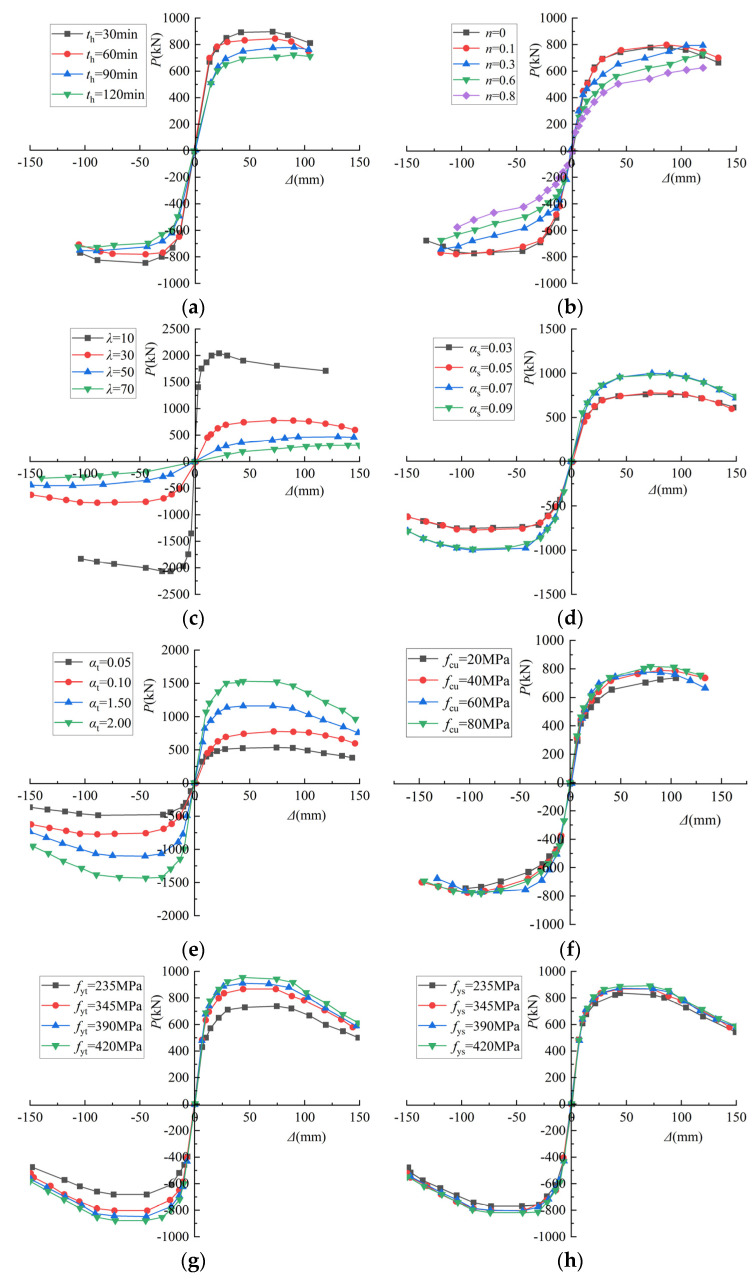

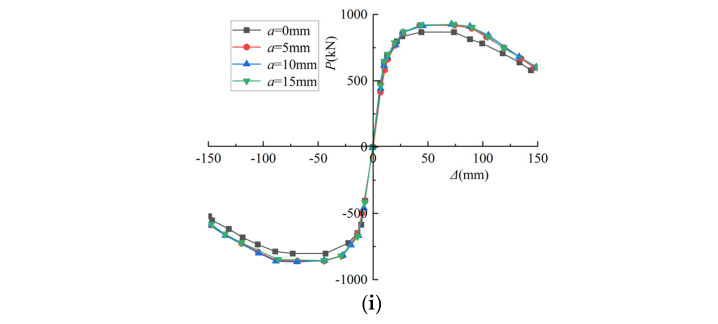
*P-Δ* skeleton curve: (**a**) Heating time; (**b**) Axial compression ratio; (**c**) Slenderness ratio; (**d**) Profiled steel ratio; (**e**) Steel tube ratio; (**f**) Concrete cubic compressive strength; (**g**) Yield strength of steel tube; (**h**) Yield strength of profiled steel; (**i**) Protective layer thickness.

Figure 18a illustrates the impact of heating time on the *P-Δ* relationship curve. It can be found that with the extension of the fire time, the horizontal bearing capacity of the SRCFST columns displayed a decreasing trend, and the stiffness of the elastic phase also decreased where the heating period was 60 min, 90 min, and 120 min, respectively. When compared to the heating period of 30 min, the positive ultimate bearing capacity decreased by 5.8%, 13.1%, and 19.4%, respectively. The horizontal ultimate bearing capacity of the component with *t_h_* = 120 min is only reduced by 7.3% compared with *t_h_* = 90 min, which is because the heating time is more than 90 min, the historical maximum overfire temperature was closed to each component, and the effect on the deterioration of material is tiny.

#### 4.1.2. Axial Compression Ratio

Figure 18b shows the effect of the axial compression ratio on the *P-Δ* relationship curve, when *n* = 0, *P*_max_ = 778.022 kN, when *n* = 0.1, *P*_max_ = 798.854 kN, when *n* = 0.3, *P*_max_ = 1000 kN. It should be said that when the axial compression ratio is low, boosting the axial compression ratio can improve the horizontal bearing capacity of the column appropriately, and the curve shows a distinctly strengthening phenomenon. When the axial compression ratio exceeds 0.3, the *P*_max_ is decreased with the increase in *n*, when the *n* = 0.8, the *P*_max_ is reduced by 40% compared with the *n* = 0.3. This is because when the axial pressure is relatively small, the concrete is cracked by tension, but the axial pressure will close the cracks, which is beneficial to the seismic resistance. When the axial load is high, the concrete is crushed, and the beneficial effect is lost, resulting in a reduction in bearing capacity.

#### 4.1.3. Slenderness Ratio

As can be seen from Figure 18c, the slenderness ratio not only affects the value of the *P-Δ* relationship curve but also affects the shape of the curve. With the addition of the slender ratio, the stiffness of the elastic phase decreased, and the horizontal bearing capacity decreased significantly. The horizontal ultimate load capacity of the columns decreased by 61.9%, 77.13%, and 84.6% when λ was 30, 50, and 70, respectively, compared to when the slenderness ratio was 10, which is similar to unfired, owing to the second-order effect. Meanwhile, the slenderness ratio affects the damage mechanism of the column members; when the slenderness ratio of the member is relatively small, the column undergoes strength damage, and when the slenderness ratio of the member is relatively large, the column undergoes instability damage due to the second-order effect.

#### 4.1.4. Profiled Steel Ratio

It can be seen from Figure 18d that the horizontal ultimate bearing capacity of *α_s_* = 0.05 was 2.2% higher than that of *α_s_* = 0.03, and the ultimate bearing capacity of *α_s_* = 0.07 was 22.2% higher than that of *α_s_* = 0.05, while the horizontal ultimate bearing capacity of *α_s_* = 0.09 was 1.4% lower than that of *α_s_* = 0.07. With the growth of the profiled steel ratio, the horizontal bearing capacity of the member improves, but the increased value is small, and this advantageous effect will not last. Since it is known from the previous analysis [50] that the contribution of concrete to bearing capacity is the greatest, steel tubes come in second, and profiled steel is the smallest in such components, increasing the profiled steel ratio will reduce the area of the core concrete, thus diminishing the contribution of concrete to bearing capacity while keeping the cross-sectional area constant.

#### 4.1.5. Steel Tube Ratio

The elastic phase stiffness and horizontal bearing capacity of the specimens improve as the steel tube ratio increases, as shown in Figure 18e, and this pattern is similar to CFST specimens in the literature [46]. The steel content in general mainly affects the value of the curve, and it has little effect on the shape of the P-relationship curve.

#### 4.1.6. Concrete Cubic Compressive Strength

Figure 18f illustrates that as the cubic compressive strength of concrete grew, so did the horizontal ultimate bearing capacity. Although the ultimate bearing capacity of *f*_cu_ = 40 MPa was 8.9% greater than that of *f*_cu_ = 20 MPa, and the ultimate bearing capacity of *f*_cu_ = 80 MPa was 12.7% higher than that of *f*_cu_ = 20 MPa, it is clear that the difference is tiny.

#### 4.1.7. Yield Strength of Steel Tube

As can be observed in Figure 18g, increasing the yield strength of the steel tube increases the horizontal ultimate bearing capacity, but the increase is not noticeable, and there is essentially no effect on the elastic phase stiffness. The *f*_yt_ values were 345 MPa, 390 MPa, and 420 MPa, respectively, and the ultimate bearing capacity rose by 17.4%, 23.3%, and 29.4% when compared to *f*_yt_, which was 235 MPa.

#### 4.1.8. Yield Strength of Profiled Steel

Figure 18h depicts the effect of profiled steel yield strength on the *P-Δ* relationship curve, and it can be seen that in the elastic phase, neither the stiffness nor the horizontal bearing capacity are affected significantly. The horizontal ultimate load-carrying capacity of *f*_ys_ = 345 MPa, 390 MPa, and 420 MPa was improved by 3.6%, 3.7%, and 6.6% over *f*_ys_ = 235 MPa, respectively. Since the contribution of steel sections to load-carrying capacity is so slight, changing the strength grade has a negligible effect on horizontal load-carrying capacity.

#### 4.1.9. Protective Layer Thickness

The horizontal bearing capacity of the component with a 5 mm thick protective layer was 17.99% higher than that of the bare column in this paper, as shown in Figure 18i. The effect of the thickness of the protective layer on the horizontal bearing capacity was slight, because with the protection of the fireproofing, the historical maximum overfire temperature of each component of the internal mating steel and steel was maintained.

### 4.2. Parameters Analysis of Ductility Coefficient

Ductility is a measure of the deformation capacity of the member under load. Reciprocal loading requires the structure to absorb a large amount of energy while producing a certain amount of deformation without damage; therefore, the member must be ductile, and it is necessary to investigate the parameters of the factors affecting ductility.

#### 4.2.1. Heating Time

**Figure 19:**
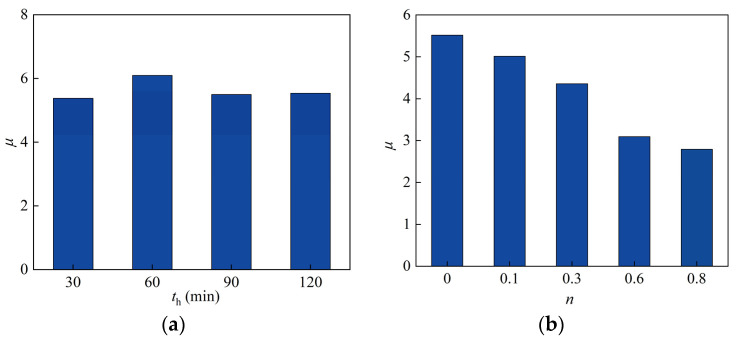

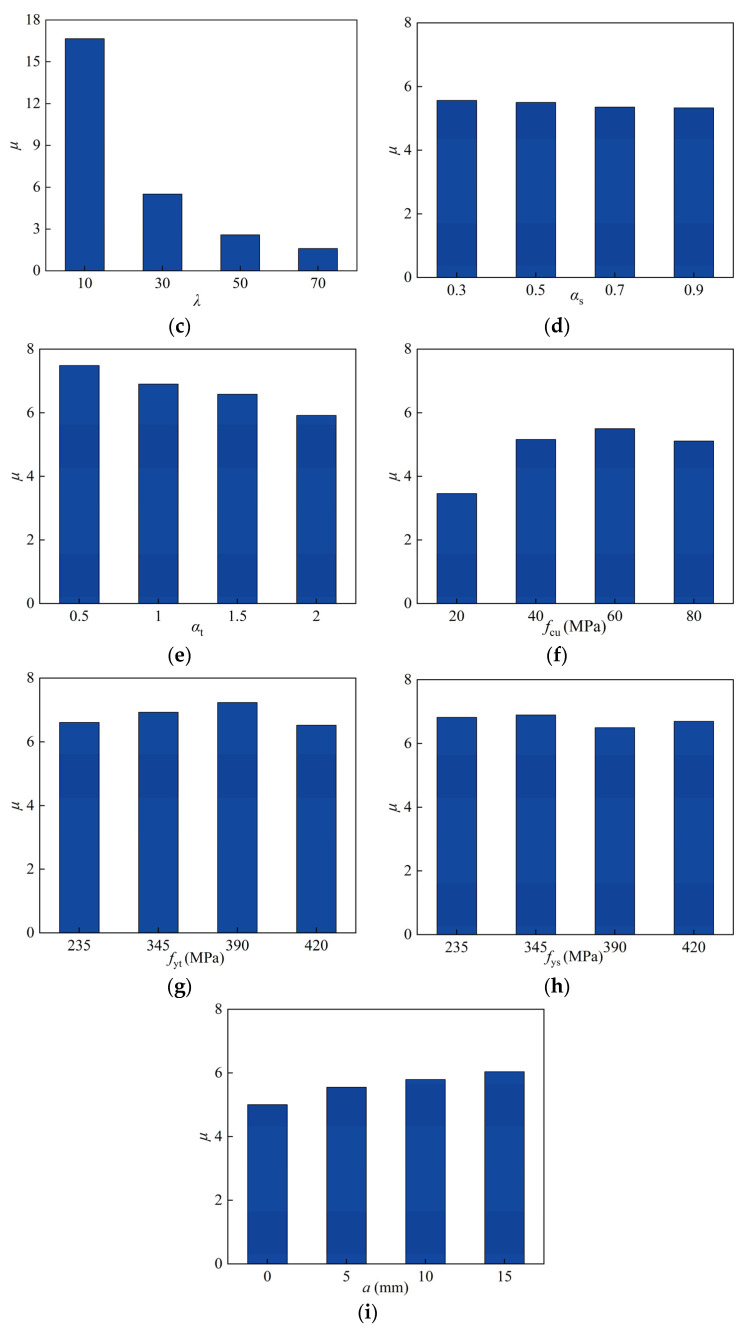
The different parameters on the ductility coefficient: (**a**) Heating time; (**b**) Axial compression ratio; (**c**) Slenderness ratio; (**d**) Profiled steel ratio; (**e**) Steel tube ratio; (**f**) Concrete cubic compressive strength; (**g**) Yield strength of steel tube; (**h**) Yield strength of profiled steel; (**i**) Protective layer thickness.

Figure 19a shows the effect of heating time on the ductility coefficient; it can be seen that the ductility coefficient of heating time of 60 min was 13.4% higher than that of heating time of 30 min. With the extension of the heating time, the ductility coefficient of the members increased firstly, because the rise of the fire time leads to the increase in the concrete compressive limit strain, resulting in the delay of concrete crushing damage, thus increasing the ductility of the specimen. However, when the fire time exceeded 60 min, the increasing trend stopped, and the ductility coefficient of the heating time of 90 min was 9.8% lower than that of heating time of 60 min. This is because the column section temperature was higher, causing material deterioration and ductility reduction. Since the historical maximum temperature and the degree of material deterioration are close, the ductility coefficient at 120 min was similar to that at 90 min.

#### 4.2.2. Axial Compression Ratio

Figure 19b depicts the effect of axial compression ratio on ductility coefficient; as the axial compression ratio was increased, the ductility coefficient tended to decrease. This regulation is similar to that observed at ambient temperature, where the axial compression ratio was 0.1, 0.3, 0.6, and 0.8, respectively. Ductility decreased by 9.1%, 21.1%, and 43.9%, respectively, when compared to when the axial pressure ratio was 0. As a result, the axial pressure ratio has a more significant impact on the ductility of this type of column after exposure to fire.

#### 4.2.3. Slenderness Ratio

The ductility coefficient of the SRCFST columns tended to decrease as the slenderness ratio increased, as shown in Figure 19c. The ductility coefficient of *λ* = 30 was 67% lower than that of *λ* = 10, the ductility coefficient of *λ* = 50 was 53.1% lower than that of *λ* = 10, and the ductility coefficient of *λ* = 70 was 37.6% lower than that of *λ* = 10, denoting that the slenderness ratio has a significant influence on the ductility coefficient.

#### 4.2.4. Profiled Steel Ratio

The effect of profiled steel ratio on the ductility coefficient is not significant, as shown in Figure 19d, for the same reason that the skeleton curve effect rule is not effective.

#### 4.2.5. Steel Tube Ratio

Figure 19e shows the influence of the steel tube ratio on the ductility coefficient. It can be noticed that as the steel tube ratio increases, the ductility coefficient decreases. Comparing *α_t_* = 1 to *α_t_* = 0.5, the ductility coefficient decreased by 7.8%; comparing *α_t_* = 1.5 to *α_t_* = 1, it decreased by 4.6%, and comparing *α_t_* = 2 to *α_t_* = 1.5, it decreased by 10.1%.

#### 4.2.6. Concrete Cubic Compressive Strength

The effect of concrete strength grade on the ductility coefficient of the SRCFST column after exposure to fire is shown in Figure 19f. It can be observed that when *f*_cu_ is less than 60 MPa, with the enhanced concrete strength grade, the ductility is improved, the ductility coefficient of *f*_cu_ = 40 MPa was 45.7% more than that of *f*_cu_ = 20 MPa, and the ductility coefficient of *f*_cu_ = 60 MPa was 7.8% more than that of *f*_cu_ = 40 MPa. However, when the concrete cubic compressive strength exceeds 60 MPa, the ductility coefficient of *f*_cu_ = 80 MPa was 7.3% lower than that of *f*_cu_ = 60 MPa.

#### 4.2.7. Yield Strength of Steel Tube

Figure 19g shows that when the yield strength was less than 390 MPa, the ductility increased slightly as the yield strength increased, but when the yield strength was greater than 390 MPa, the ductility decreased. When *f*_yt_ = 420 MPa, the ductility coefficient was reduced by 9.7% compared with when *f*_yt_ = 390 MPa. Overall, the yield strength of the steel tube has a minor impact on the ductility.

#### 4.2.8. Yield Strength of Profiled Steel

Figure 19h shows the regulation of influence for the yield strength of profiled steel on the ductility coefficient of SRCFST members after fire, which was seen to have a small effect.

#### 4.2.9. Protective Layer Thickness

Figure 19i illustrates the effect of the protective layer thickness on the ductility coefficient of this type of specimen after exposure to fire; it can be noted that the ductility coefficient of the bare column was the lowest; when the protective layer thickness was 5 mm, 10 mm, and 15 mm, the ductility coefficient increased by 11%, 15.9%, and 20.8%, respectively, when compared to the bare column.

## 5. Conclusions

Numerically simulations of the cyclic loading of SRCFST columns after exposure to fire were presented in this paper. This study’s findings can be summarized as follows:

(1) The temperature field of the SRCFST column is symmetrically distributed along the diameter, increasing the closer to the center of the circle. In addition, the concrete is thermally inert and functions as a natural fire protection layer for the profile steel, lowering the temperature of the profiled steel.
(2) Although the peak load, ductility coefficient, energy dissipation capacity, and stiffness of the skeleton line of this column were slightly reduced after fire compared to the SRCFST members at ambient temperature, the hysteresis curves did not pinch significantly and still exhibited better seismic performance.
(3) After exposure to fire, the effects of heating time (*t_h_*), axial compression ratio (*n*), slenderness ratio (*λ*), and steel tube ratio (*α_t_*) on the skeleton line of SRCFST columns are more significant. Moreover, the axial compression ratio (*n*), slenderness ratio (*λ*), and steel tube ratio (*α_t_*) have a negative influence on the ductility of SRCFST columns after fire.
(4) The hysteresis curve and stiffness of the SRCFST column after fire are similar to that of the CFST column when the total steel ratio is kept constant, while the ductility is better. To fully exploit post-fire the seismic performance of SRCFST members, the appropriate percentage of steel tube and profiled steel needs to be researched further.

## Figures and Tables

**Table 1:** List of test specimen parameters for hysteretic behavior of CFST.

Section Type	Specimen	*D* (*B*) × *t*_s_ (mm)	*t* (min)	*L* (mm)	*N*_0_ (kN)	*n*_0_
circular	CF1	133 × 4.7	90	1500	0	0
	CF2	133 × 4.7	90	1500	80	0.15
	CF3	133 × 4.7	90	1500	160	0.3
	CF5-1	133 × 4.7	90	1500	240	0.45
square	SF1	120 × 2.9	90	1500	0	0
	SF2-1	120 × 2.9	90	1500	60	0.15

**Table 2:** Detailed information of SRCFST column.

Section Type	Specimen	*D* × *t*_s_ (mm)	*t_h_* (min)	*α_t_*	*α_s_*	*λ*	*f*_ys_ (MPa)	*f*_yt_ (MPa)	*f*_cu_ (MPa)	*n*
circular	SRCFST	400 × 9	90	0.1	0.05	30	345	345	60	0.6

*D* is the diameter of the section, *t_s_* is the thickness of the steel tube, *t_h_* is the heating time, *α_t_ = A_t_*/*A_c_* (*A_t_* is the section area of the steel tube, *A_c_* is the section area of the concrete) is the steel tube ratio, *α_s_ = A_s_*/*A_c_* (*A_s_* is the section area of the profiled steel) is the profiled steel ratio, *λ* = 4 *L*/*D* is the slenderness ratio, *f*_ys_ is the yield strength of the profiled steel, *f*_yt_ is the yield strength of the steel tube, *f*_cu_ is the concrete cubic compressive strength, and *n* is the axial compression ratio.

**Table 3:** The results of ductility.

Specimen	Direction	Yield Load *P*_y_/kN	Yield Displacement *Δ*_y_/mm	Maximum Load *P*_max_/kN	Maximum Displacement *Δ*_max_/mm	Failure Displacement *Δ*_u_/mm	Ductility *μ*
Post-fire	+	729.9	49.2	907.9	120.0	120.0	2.4 1.8
	−	910.2	103.3	1019.2	119.7	119.7	1.2
Ambient temperature	+	891.8	27.4	1090.8	125.0	132.8	4.5 3.1
	−	1040.1	81.2	1259.9	134.2	134.2	1.6

**Table 4:** Comparison of ductility coefficients.

Specimen	Direction	Yield Load *P*_y_/kN	Yield Displacement *Δ*_y_/mm	Ultimate Load *P*_max_/kN	Ultimate Displacement *Δ*_max_/mm	Failure Displacement *Δ*_u_/mm	Ductility *μ*
SRCFST	+	64.8	20.2	76.1	79.5	77.4	3.8 3.7
	−	64.7	21.2	76.1	80.1	80.1	3.5
CFST	+	71.8	19.2	91.8	80.2	80.2	4 3.4
	−	69.6	18.4	85.8	61.5	61.5	2.8

**Table 5:** Summary of specimen dimensions of the post-fire seismic performance.

Parameter	Values	Default Values
Heating time *t_h_*/(min)	30, 60, 90, 120,	90
Axial compression ratio *n*	0, 0.1, 0.3, 0.6, 0.8	0
Slenderness ratio *λ*	10, 30, 50, 70	30
profiled steel ratio *α_s_*	0.03, 0.05, 0.07, 0.09	0.05
Steel tube ratio *α_t_*	0.05, 0.10, 0.15, 0.20	0.1
Concrete cubic compressive strength *f*_cu_/(N/mm^2^)	20, 40, 60, 80	60
Yield strength of steel tube *f*_yt_/(N/mm^2^)	235, 345, 390, 420	345
Yield strength of profiled steel *f*_ys_/(N/mm^2^)	235, 345, 390, 420	345
Protective layer thickness *a/*(mm)	0, 5, 10, 15	0

## Data Availability

The data presented in this study are available on request from the corresponding author.

## Nomenclature

*A_c_*	section area of concrete
*A_s_*	section area of profiled steel
*A_t_*	section area of steel tube
*B*	width of concrete-filled square steel tube column
*D*	diameter of circular SRCFST column
*E*	energy dissipation
*K_j_*	secant stiffness
*λ*	slenderness ratio of column
*α_t_*	steel tube ratio
*α_s_*	profiled steel ratio
*t_h_*	heating time
*t_s_*	thickness of the steel tube
*t*_w_	width of profile web
*t*_f_	width of profile flange
*f*_yt_	yield strength of steel tube
*f*_cu_	concrete cubic compressive strength
*L*	length of the specimen
*n*	axial compression ratio of column
*a*	protective layer thickness
*μ*	ductility coefficient of column
*Δ*_u_	failure displacement
*Δ*_y_	yield displacement
*Δ*_max_	ultimate displacement
*Δ*_j_	displacement corresponding to *P_j_* under the first cycle of level *j*
*P*_y_	yield load
*P*_max_	ultimate strength
*P*_u_	failure load
*P_j_*	peak load under the first cycle of level *j*
